# New insights on circumscribed choroidal hemangioma: “bench to bedside”

**DOI:** 10.1007/s00417-023-06179-x

**Published:** 2023-07-28

**Authors:** Marco Lupidi, Chiara Centini, Greta Castellucci, Michele Nicolai, Nicola Lassandro, Carlo Cagini, Clara Rizzo, Jay Chhablani, Cesare Mariotti

**Affiliations:** 1 Eye Clinic, Department of Experimental and Clinical Medicine, Polytechnic University of Marche, Ancona, Italy; 2 Fondazione Per La Macula Onlus, Dipartimento Di Neuroscienze, Riabilitazione, OftalmologiaGenetica e Scienze Materno-Infantili (DINOGMI), University Eye Clinic, Genoa, Italy; 3 Department of Medicine and Surgery, University of Perugia, S. Maria Della Misericordia Hospital, Perugia, Italy; 4 Ophthalmic Unit, Department of Neurosciences, Biomedicine, and Movement Sciences, University of Verona, Verona, Italy; 5 Department of Ophthalmology, UPMC Eye Center, University of Pittsburgh, Pittsburgh, USA

**Keywords:** Choroidal hemangioma, Circumscribed choroidal hemangioma, Subretinal fluid, OCT, Indocyanine green angiography, Photodynamic therapy

## Abstract

Circumscribed choroidal hemangioma is a rare vascular hamartoma of the choroid, presenting as a red–orange mass at the posterior pole on fundoscopic examination. Despite its benign origin, associated complications such as subretinal fluid, serous retinal detachment, retinoschisis and neovascular glaucoma may lead to serious visual impairment in more than half patients. Because of its similarity to amelanotic choroidal melanoma and choroidal metastasis, differential diagnosis is still challenging for specialists. Multimodal imaging such as ultrasonography, fluorescein angiography, indocyanine green angiography, optical coherence tomography, and optical coherence tomography angiography guides the clinician to the correct diagnosis and the proper follow-up. Treatment is indicated in symptomatic cases in order to resolve exudation and improve visual acuity. Treatment options include photocoagulation, transpupillary thermotherapy, radiation therapy, photodynamic therapy and anti-vascular endothelial growth factor therapy. Currently, photodynamic therapy is the treatment of choice due to its effectiveness and safety. The purpose of this review is to describe the latest knowledge in the etiopathogenesis of the circumscribed choroidal hemangioma, the most recent multimodal imaging findings, and the available treatment options.


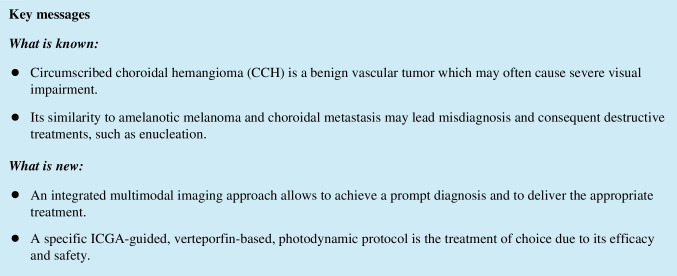


## Introduction

The choroidal hemangioma (CH) is an uncommon benign vascular tumor of the choroid which can occur in a circumscribed or in a diffuse form. The circumscribed choroidal hemangioma (CCH) is sporadic and normally it is not associated with systemic manifestations [1–3]. Even though it is usually diagnosed in the fourth to sixth decades, the congenital origin is not excluded [1]. It shows an higher incidence in males and Caucasians. Usually it is solitary and unilateral, but two cases of multifocal CCH [4, 5] and rare bilateral cases [6–8] have been documented. The diffuse choroidal hemangioma (DCH) represents a clinical feature of half patients affected by Sturge-Weber syndrome [9]. Symptoms typically occur within adolescence, even though it is present since birth. This condition is usually unilateral, but it was reported a rare bilateral case [10].

In the current review we described the etiopathogenesis of the CCH, its clinical features and complications, the recent multimodal imaging findings, the main differential diagnoses, and the available treatment options.

## Clinical and histological features

Circumscribed and diffuse CHs present clinical and histological differences. CCHs are located at the posterior pole, mostly in the supero-temporal quadrant close to the macula, and show well-defined margins formed by a layer of compressed melanocytes and choroidal lamellae [5, 11]. Whereas DCHs usually involve over half of the choroid and show ill-defined borders blending imperceptibly into the peripheral choroid. The uninvolved choroid shows engorged large vessels and also episcleral and subconjunctival perilimbal tissues exhibit large numbers of extremely dilated, thin-walled blood vessels [11].

Histologically, a CCH can be formed by capillary, cavernous or both types (mixed type) of blood vessels and the choriocapillaris is never primarily involved by the vascular growth [11]. The capillary type exhibits small capillary vessels with a single layer of flat endothelial cells divided by loose edematous connective tissue, whereas the cavernous type shows large thin-wall vessels also delineated by a flat endothelium and separated by thin intervascular septa. Both types have no smooth muscle cells in the vessels wall, but aggregates of leukocytes are present, especially neutrophils and eosinophils. Usually, the CCH is represented by the cavernous type, while the DCH associated with Sturge-Weber syndrome is typically represented by the mixed type [11].

The masses probably originate from hemodynamic turbulences such as persistent arteriovenous shunts which did not disappear during embryogenesis, as it normally occurs [11]. Differently from other choroidal tumors, CHs are non-proliferative lesions with little or no tendency to enlarge. When enlargement occurs, the probable prime mover is venous congestion [12]; this hypothesis is validated by a reported case of tumor enlargement during pregnancy and decrease after delivery [13]. Another cause may be a slow growth in the caliber and number of the intra-tumoral vessels, as it is commonly described in the cavernous hemangioma of the orbit [14].

Thin-wall vessels formed by a single endothelium layer and hemodynamic turbulences are probably responsible of CH’s complications such as exudation. Lee et al. [15] investigated for the first time different patterns of retinal fluid accumulation related to CCH in patients with visual symptoms, treatment-naïve and followed-up for more than 2 years. They found out that subretinal fluid (SRF) was the main pattern at initial presentation (73.1%), while SRF associated with intraretinal fluid (IRF) was present in the 15.4% of cases and advanced cystoid macular edema (CME) in the 11.5%. Over time, the pattern changed from SRF only to SRF and IRF combined, and advanced CME was mainly observed in long-term follow-up regardless treatment modality, suggesting a long-standing disease refractory to different treatment. The pathophysiology of CME in CCH is still not completely understood. CME often occurs due to breakdown of the inner blood–retina barrier, but long standing SRF may induce alteration of external limiting membrane (ELM), allowing the passage of fluid into the outer retina [15].

## Diagnosis

The diagnosis is primarily ophthalmoscopic and it can be incidental or secondary to symptoms like blurred vision, metamorphopsia, floaters and field defects. When the location is subfoveal, a hyperopic shift and secondary refractive amblyopia can appear. In more than half cases, patients experience visual loss with a visual acuity (VA) of 20/200 or worse because of complications such as SRF, serous retinal detachment, secondary retinal pigment epithelium (RPE) changes such as hyperplasia or metaplasia, retinoschisis and neovascular glaucoma [1]. Longer symptoms duration, previous failed treatment, lower initial VA, and presence of retinoschisis are risk factors for worse visual outcomes [16].

At the funduscopic examination, CCH appears as a round or oval orange-red subretinal mass similar to the adjacent choroid but separated from the latter by a ring of slight pigmentation, as a result of compressed melanocytes and choroidal lamellae [11]. The mass itself is nonpigmented, mimicking amelanotic melanoma and choroidal metastasis, but it can show overlying pigmentation over time. The main location is posterior to the equator, usually within one to three disk diameters from the macula [11]. The height is typically less than 5 mm and rarely exceeds 6 mm. The tumor generally presents a diameter to height ratio > 2 [5]. A low tumor diameter/height-ratio and a short distance between the tumor and the optic disc were found to be significant risk factors for exudation [5]. The overlying RPE is often involved, spreading from mild atrophy to focal proliferation with drusen formation to severe fibrous transformation. Different stages of obliteration and sclerosis are present in areas overlying the tumor, with severe chorioretinal scarring and ossification correlated to disease’s duration. Loss of photoreceptors, severe cystoid degeneration of the outer layers and marked gliosis are described. The ganglion cells and the nerve fibers layers are generally well preserved, except for cases with longstanding glaucoma [11].

Anterior segment examination is usually unremarkable. Rarely, dilatated episcleral vessels (4%), iris neovascularization (1%) and heterochromia iridis (1%) have been described [1]. The intraocular pressure is normal, except for patients with neovascular glaucoma secondary to extensive retinal detachment [11].

## Imaging modalities

Despite quite typical clinical features, the CCH diagnosis is still a challenge for specialists. Only one third of patients seems to be referred with the correct diagnosis [1, 5]. Its similarity to amelanotic melanoma and choroidal metastasis may lead to misdiagnosis and consequent destructive treatments, such as enucleation. Thanks to the advent of new imaging techniques, the use of ancillary tests guides the clinician to the correct diagnosis.

### Ultrasound sonography

The ultrasound sonography (USG) is an important adjuvant examination for the clinical assessment of the CCH as it provides a simple, rapid, and dynamic study of the posterior segment. It is helpful in measuring the mass dimension and monitoring tumor progression (with and without treatment). CCH shows high internal reflectivity on A-scan due to the multiple vascular channels inside the tumor. On B-scan, it appears as a dome-shaped moderately elevated choroidal mass (with a mean diameter of 6.7 mm, a mean height of 2.2 mm and a median basal diameter/height ratio of 3) with an acoustic solidity similar to the healthy surrounding choroid. Choroidal excavation is seen in about a fifth of cases [1, 5, 17–19].

### Autofluorescence

Autofluorescence (AF) is a useful tool in the evaluation of CCH history (previous treatments, exudation chronicity) and of the status of the overlying RPE. CHs not previously treated show intrinsic iso-AF or hypo-AF, while treated CHs are most likely to appear hypo-autofluorescent. Tumor location, size, or thickness do not correlate with intrinsic AF pattern. Recent SRF with intact RPE is typically hyper-AF. In contrast, RPE fibrous metaplasia and RPE atrophy or hyperplasia are moderately to markedly hypo-AF. The brightest hyper-AF is associated with orange pigment overlying the lesion [20].

### Fluorescein angiography

**Fig. 1:**
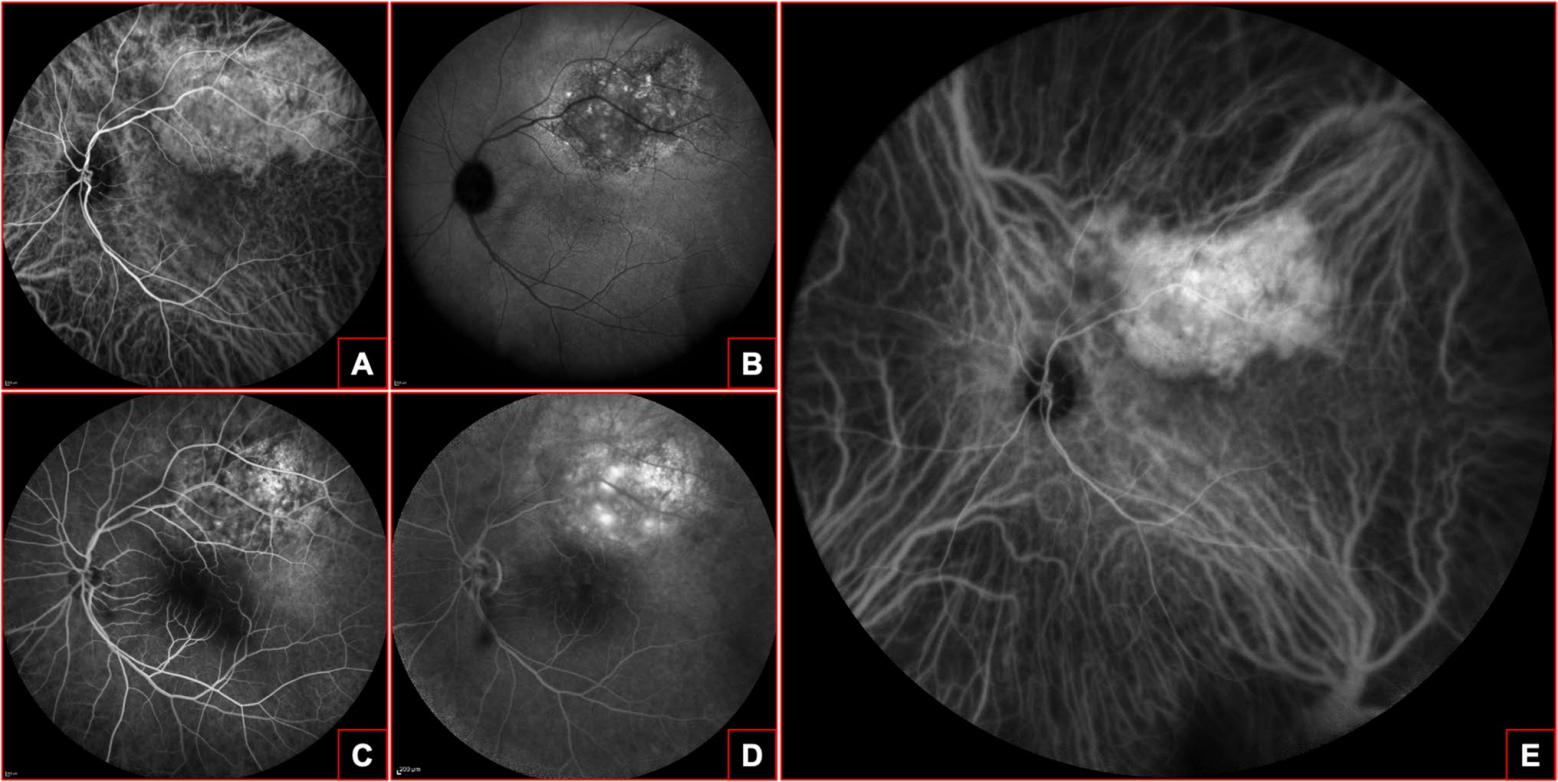
Circumscribed choroidal hemangioma (CCH). **A**, early venous phases of a 55° indocyanine green angiography (ICGA) showing an extensive CCH (4-disc diameters in the greatest linear diameter) located in the supero-temporal quadrant of the posterior pole. **B**, 55° ICGA during the inversion phases revealing the typical late dye wash-out of the CCH. **C**—**D**, early and late fluorescein angiography of the previously described CCH revealing the retinal pigmented epithelium impairment associated with the outer blood-retinal barrier decompensation (leakage). E, ultra-wide-field ICGA (102°) revealing the entire CCH and its topographical relationship with the vortex veins

Fluorescein angiography (FA) displays CH as an early mild hyperfluorescent lesion in the prearterial (choroidal filling phase) and early arterial phases, followed by moderate hyperfluorescence during the arteriovenous phase and increasing hyperfluorescence and progressive staining of the extravascular tissue of the mass with variable leakage in the late phase (Fig. 1). Unfortunately, FA is not pathognomonic because almost all choroidal tumors have the same angiographic features but combined with other imaging findings it supports the clinician in making the correct diagnosis and treatment planification. Indeed, FA allows to visualize CCH limits, as well as the extent of complications such as CME and serous retinal detachment and areas of leakage that may need retreatment [5, 12, 19, 21].

### Indocyanine green angiography

Indocyanine green angiography (ICGA) shows an immediate hyperfluorescence due to a rapid filling of the intratumoral vessels, in contrast with the hypofluorescent tumoral background where normal choroidal vessels are absent (arterial phase). Subsequently, the lesion shows a patchy hyperfluorescence on which even more strongly hyperfluorescent points are superimposed (venous phase). These hyperfluorescent points are the only hyperfluorecent areas during the very late phase (20 min), in which a relative hypofluorescence (compared to the adjacent choroid) with a perilesional hyperfluorescent ring is observed [5, 22–24] (Fig. 1). This angiographic finding is called “washout phenomenon” and is pathognomonic of CH. ICGA is an essential tool to visualize CH vasculature. This technique is superior to FA in detecting intrinsic vessels of the hemangioma, as well as in differentiating the CCH from other amelanotic choroidal tumors.

### Optical coherence tomography

**Fig. 2:**
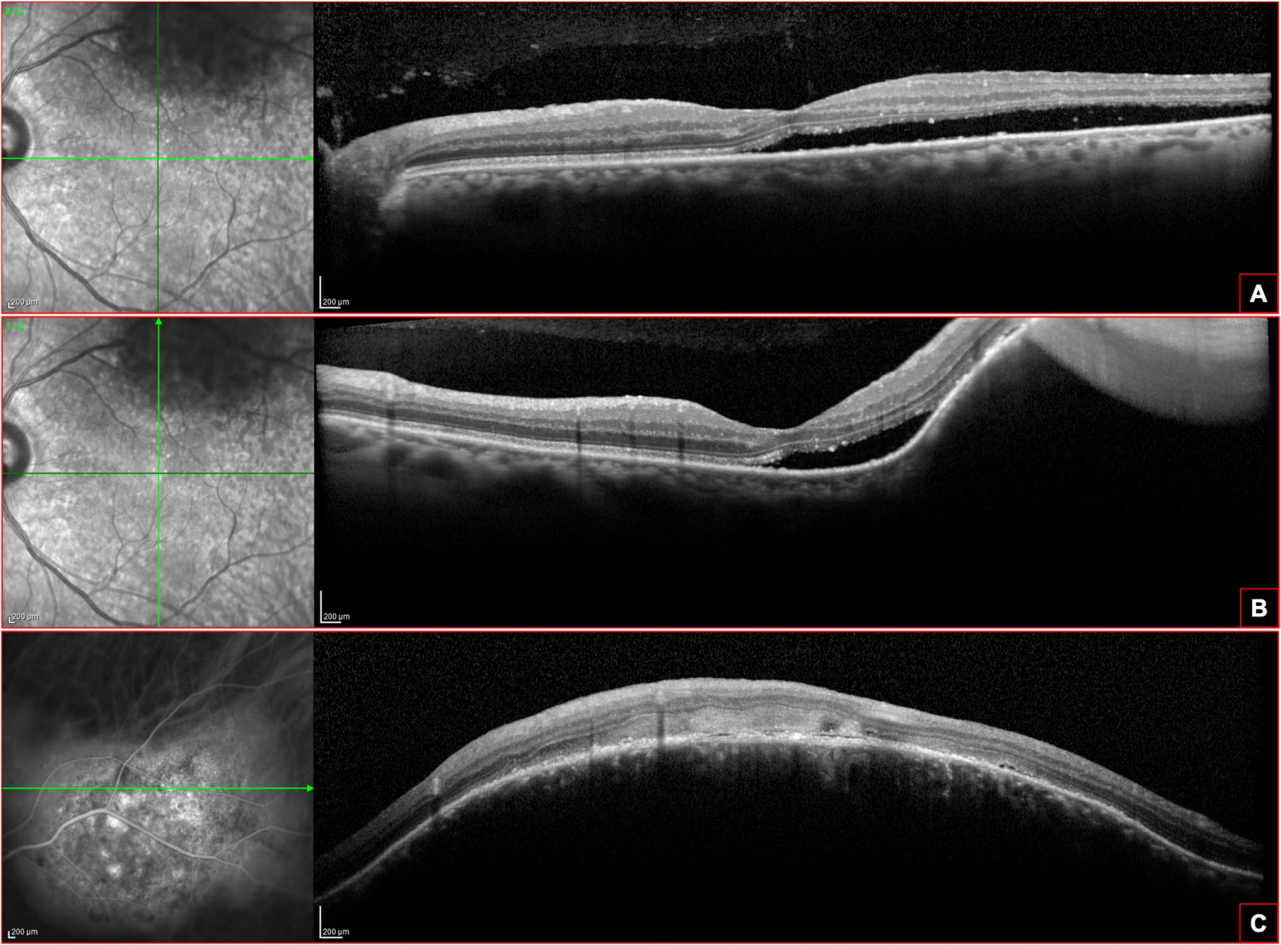
Optical coherence tomography (OCT) of a circumscribed choroidal hemangioma (CCH). **A**, horizontal structural-OCT scan revealing a retinal neurosensory detachment of the temporal macula with foveal involvement. **B**, vertical structural-OCT scan showing a “dome-shaped” retinal pigmented epithelium (RPE) elevation associated with a limited sub-retinal fluid accumulation in foveal area. **C**, the structural-OCT at the apex of the CCH is characterized by diffuse RPE abnormalities with focal hyper-transmission, loss of the photoreceptors layer and subretinal hyper-reflective material with minimal serous accumulation

Spectral domain-optical coherence tomography (SD-OCT) finds its primarily application in detecting and monitoring CCH complications (Fig. 2), such as CME or SRF. Moreover, this imaging technique is an useful tool to assess treatment efficacy and to evidence retinal changes associated with CCH, such as RPE alterations.

On SD-OCT images, CCH appears as an abrupt dome-shaped hyporeflective choroidal mass with the expansion of medium- and large-size choroidal vessels, without compression of the choriocapillaris, and intact Bruch’s membrane. CCH and small amelanotic choroidal melanoma share similar tomographic features, but choroidal vessels are compressed in melanoma, differently from CCH [25, 26]. SD-OCT is less resolutive in the differential diagnosis of vascular tumors and it is able to evaluate only posterior lesions from the retina to the inner choroid.

Enhance depth imaging-optical coherence tomography (EDI-OCT) enables the clinician to acquire high-resolution images of the choroid, overcoming the relatively low-resolution imaging of the choroid and the sclera obtained with standard OCT due to the light scattering generated from the RPE and the choriocapillaris. On EDI-OCT scans, CCH is depicted as a medium to low reflective lesion with homogeneous signal and intrinsic spaces, probably corresponding with vascular spaces [27].

EDI-OCT seems to be more accurate than other imaging techniques such as conventional USG in estimating hemangioma’s dimensions, especially for lesions < 1 mm. Indeed, USG may overestimate tumor thickness because of the difficulty in pinpointing the posterior choroidal margin, the poorer resolution of the overlying retina and the gross estimation using USG calipers [24]. In agreement with this assumption, Ozkurt et al. [28] found that CCH thickness measured with EDI-OCT was 37% inferior to the one quantified with USG. Similarly, Rojanaporn et al. [25] evaluated treatment naïve CCHs features using EDI-OCT in ten eyes of ten Caucasian patients (3 men and 7 females) with a mean age of 50. They reported a mean tumor diameter of 5.4 mm and a mean tumor thickness of 1.187 mm, a 50.5% reduction compared with 2.4 mm obtained with USG. Other EDI-OCT findings included partial choroidal shadowing (90%), intact Bruch’s membrane (100%), absence of choriocapillaris compression (100%), expanded medium and large size choroidal vessels without compression (100%), visible tumor interface and lateral “pushing” margins (100%). Medium vessels within the hemangioma shown a mean expansion of 265% compared to those in the healthy choroid, while large vessels displayed a mean expansion of 576% compared to the normal ones [25].

### OCT-angiography

OCT-angiography (OCTA) is a non-invasive imaging technique useful to identify and monitor many retinal diseases without the use of dye. It represents a promising tool for the diagnosis and follow-up of choroidal tumors such as CCH because it allows an adequate visualization of both choroidal and tumoral vascularization.

**Fig. 3:**
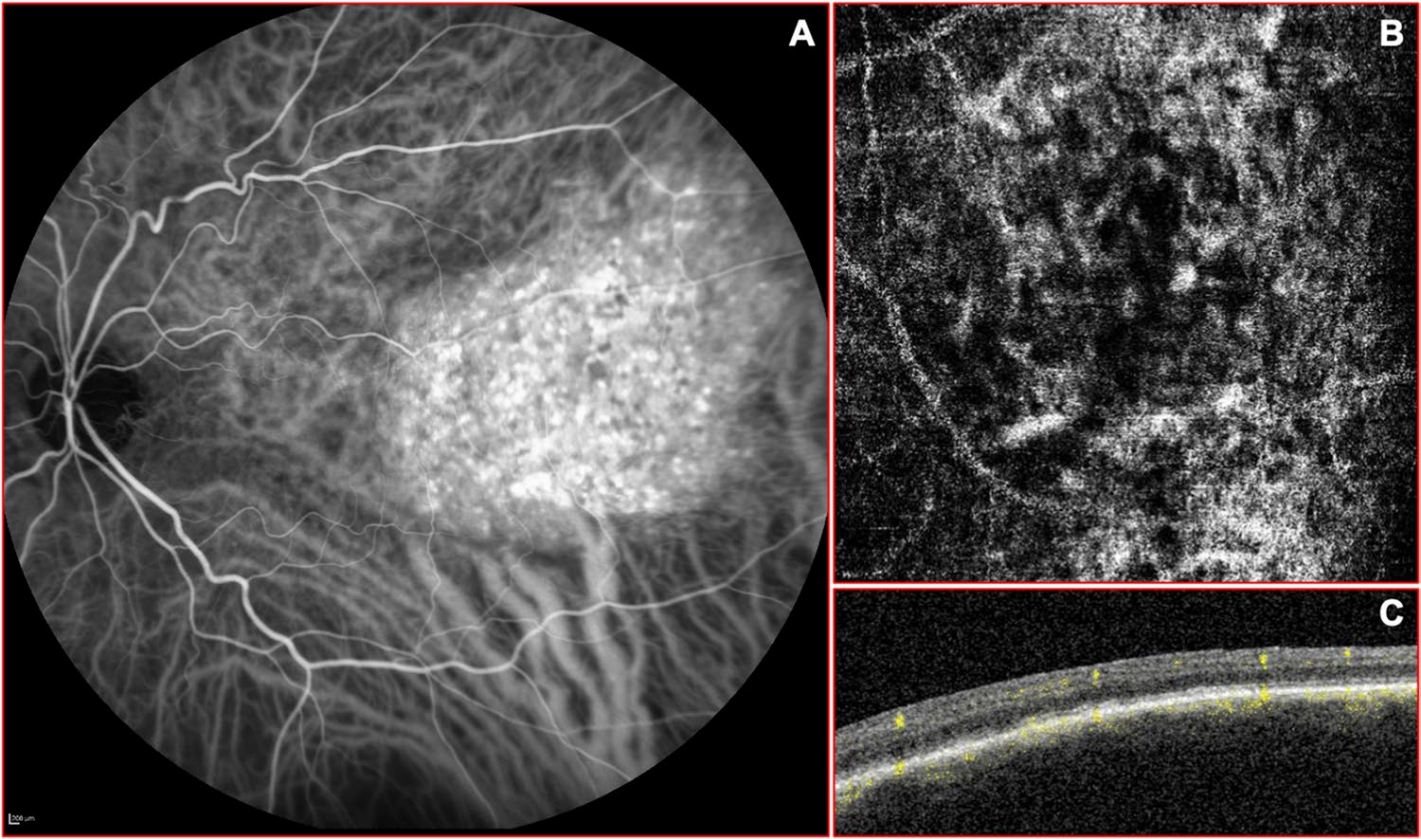
Optical coherence tomography-angiography (OCT-A) of a circumscribed choroidal hemangioma (CCH). Reference ICGA image of CCH (**A**). The en-face visualization of the OCT-angiogram (**B**) shows a perfused capillary network of variably sized, dilated and disorganized vessels, compared to the surrounding choriocapillaris. The CCH vessels are separated by intervascular septa where flow is not detected with rarefied vessels at the periphery of the lesion. The focal lacks of flow are clearly visible on the B-scan visualization of the lesion (**C**)

On OCTA, CCH appears as a richly branched network of variably sized interconnected vessels, larger than normal surrounding choroidal vessels in both superficial and deeper choroidal slabs, and separated by intervascular septa (Fig. 3). Internal tumor vessels are pathognomonic and have been described with a variety of names including “club-shaped”, “bag of worms” and “spaghetti-like”. Gönen et al. [29] reported four vascular patterns at the level of the choriocapillaris: “bag of worms”, “club-like appearance”, giant choroidal vessels and normal choriocapillaris. The rates of these vascular patterns were 40%, 30%, 10%, and 20% in treatment-naïve CCH and were 46.1%, 30.8%, 7.7%, and 15.4% in photodynamic therapy (PDT)-treated CCH, respectively. Tumor margins show a distinct peripheral circumferential arcade of rarefied vessels with radially projecting branches [30, 31]. CCHs generally do not involve parafoveal inner retinal microvasculature, but a reduction in deep capillary plexus density can be expected in eyes with previous or current CME or SRF. Associated neovascular membranes are usually absent [32]. In a case report, Takkar et al. [30] described the presence of dark areas inside CCH, whose correct interpretation needs further investigation, as they may be a consequence of increased retinal pigment in these areas, turbulent flow, blood flow velocity outside the detected range or shadow artifacts. Shanmugam et al. [33] observed dark areas as well, hypothesizing they may be a consequence of blood flow dynamics changes in the tumor vessels. CCH blood flow pattern may influence tumor stability and exudation, as a “club-like” appearance in deeper choroidal layers is more common in active tumors, while signal void areas in deeper choroidal slabs are prominent in stable tumors, probably representing connective tissue portions of the hemangioma [30, 33].

Swept- source OCT (SS-OCT) uses a longer wavelength (1050 nm) and a higher scan speed, resulting in a great tissue penetration and a better visualization of the internal architecture of the vascular tumors compared to SD-OCT system. On en face SS-OCTA images, CCH presents a well-delimitated, multilobular pattern (honeycombing-like), well distinguished from the surrounding choroid. The hemangioma shows hyporeflective, confluent oval or round areas corresponding with the lumen of the tumor vascular spaces, and hyper-reflective zones, which may depict the vessels walls and connective tissue of the tumor [30, 33, [34]. A hyper-reflective halo surrounding the tumor was found in some patients and it may represent the accumulation of choroidal melanocytes and choroidal lamellae from the healthy choroid described by Witschel and Font [11]. No differences between naïve patients and treated-patients have been described [35].

Despite its limitations, especially the inability to detect tumors located beyond the vascular arcades and the risk of partial visualization of lesions of major dimensions, OCTA is a rapid, high resolution technique and it is useful in monitoring vascular changes and so treatment response after therapy, as brachytherapy, PDT and transpupillary thermotherapy (TTT) [36].

## Differential diagnoses

Despite all the ancillary tests available, the diagnosis of CCH is still a challenge. According to Shields et al. [1], out of 200 cases of CCH, only 29% of them were correctly detected. The most common referral diagnoses were choroidal melanoma (29%), choroidal metastasis (9%), retinal detachment (6%) and central serous chorioretinopathy (CSCR) (5%). Approximately 14% of cases did not receive a diagnosis. It is important to differentiate the CCH from malignant affections because of their morbidity, mortality, and demand for an invasive and destructive management. The main differential diagnoses of CCH are summarized on Table 1.

**Table 1:** Differential diagnosis of circumscribed choroidal hemangioma

	CHOROIDAL HEMANGIOMA	CHOROIDAL MELANOMA	CHOROIDAL METASTASIS	CENTRAL SEROUS CHORIORETINOPATHY
Dilated funduscopic exam (DFE)	Round or oval orange-red subretinal mass similar to the adjacent choroid The mass itself is nonpigmented, but it may show overlying pigmentation over time	Well- circumscribed pigmented dome- shaped nodular mass, with black or grey appearance Amelanotic melanomas (15% of cases): frequent misdiagnosis. Yellow to tan in color with overlying drusen in some cases	Creamy white or pale-yellow mass associated with SRF Orange in color in thyroid carcinoma, renal cell carcinoma and carcinoid tumor metastases, mimicking CCH	Serous retinal detachment, typically in working-age men
Localization	Posterior to the equator, usually within one to three disk diameters the macula	Anywhere in the posterior segment	Multifocal and bilateral in 15–50% of cases	
Ultrasound sonography (USG)	High internal reflectivity on A-scan On B-scan dome-shaped moderately elevated choroidal mass and acoustic solidity similar to the healthy surrounding choroid Height typically < 5 mm	Acoustic hollowness on A-scan Moderate to low internal reflectivity on B-scan A mass with height > 3 mm and length > 2 mm is suggestive of choroidal melanoma	Similar to CCH, but often multilobular with an irregular surface and a diffuse infiltration of the surrounding choroid	
Autofluorescence (AF)	Hypo/iso-AF	Confluent hyper-AF		Areas of granular hypo-AF and mixed stippled hyper- and hypo-AF
Fluorescein angiography (FA) and Indocyanine green angiography (ICGA)	FA: early mild hyperfluorescence in the early arterial phase, moderate hyperfluorescence during the arterio-venous phase and increasing hyperfluorescence with variable late leakage in the late phase ICGA: “washout phenomenon” = early hyperfluorescence followed by a relative hypofluorescence. Pathognomonic of CCH	Slower and less intense filling compared to CCH. Hypofluorescent on ICGA (NP)	ICGA: larger area of choroidal involvement than on clinical examination or FA. (NP)	FA: hyperfluorescent areas in all phases. RPE leakage is described with a “smoke-stack” or an “ink-blot” pattern ICGA: dilated choroidal vessels and choroidal filling defects in early phase, with mid-phase indistinct hyperfluorescence and late staining
Magnetic resonance imaging (MRI)	Hyperintensity on T1- and iso-hyperintensity on T2-weighted images	Isointensity on T1- and hypointensity on T2-weighted images	Isointensity on T1- and hypointensity on T2-weighted images	
Optical coherence tomography (OCT)	Hyporeflective choroidal mass with the expansion of medium-and large-size choroidal vessels, without compression of the choriocapillaris (NP)	Compressed choroidal vessels. Higher reflectivity of SRF compared to CCH and choroidal metastasis	Irregular or “lumpy bumpy” anterior surface of the lesions	SRF, PEDs, double-layer sign, vacuole sign, hyperreflective dots in the neuroretina and subretinal space EDI-OCT: increased choroidal thickness and choroidal vessels dilatation
OCT-angiography (OCT-A)	Dense irregular vascular network in CCL and ORL	Dense irregular vascular network in CCL and ORL	Lack of flow at the level of the lesion and absence of pathological blood flow in the ORL	

*DFE* = dilated funduscopic exam; *USG* = Ultrasound sonography; *FA* = Fluorescein angiography; *ICGA* = Indocyanine green angiography; *MRI* = Magnetic resonance imaging; *OCT* = Optical coherence tomography; *OCT-A* = OCT-angiography.

*NP* = non pathognomonic; *SRF* = sub-retinal fluid; *AV* = arteriovenous; *CCL* = choroid capillary layers; *ORL* = outer retinal layers

### Choroidal melanoma

Choroidal melanoma is the most common primary intraocular malignant tumor. On fundus examination, choroidal melanoma typically appears as a well-circumscribed pigmented dome-shaped nodular mass with black or grey appearance. This lesion may be detected anywhere in the posterior segment of the eye. Choroidal melanoma often has a mushroom shaped appearance, almost never described in CCH. Amelanotic melanoma is detected in 15% of cases and may be misdiagnosed with CCH, although the lesion is typically different, presenting its yellow to tan in color with overlying drusen in some cases. On USG, choroidal melanoma exhibits moderate to low internal reflectivity on B-scan and acoustic hollowness on A-scan. It may present a dome (75%), mushroom (20%) or diffuse (5%) configuration and choroidal excavation is described in two-third of cases. A mass with height > 3 mm and length > 2 mm is suggestive of choroidal melanoma. [37] AF may be useful in differentiating choroidal melanoma from CCH as melanoma shows confluent hyper-AF in contrast with the hypo/iso-AF of CCH [38]. FA and ICGA are not pathognomonic, but the choroidal melanoma has a slower and less intense filling compared to the CCH and appears hypofluorescent on ICGA. On MRI images, the choroidal melanoma is isointense on T1- and hypointense on T2-weighted images, whereas the CCH shows T2 iso-hyperintensity. A choroidal mass located entirely posterior to the equator of the globe and with intense enhancement on MRI study is likely to be a CCH [39, 40]. Differently from CCH, choroidal vessels appear compressed on OCT scans in case of choroidal melanoma and the associated SRF has higher reflectivity compared to CCH and choroidal metastasis [41]. On OCTA images, both lesions present a dense irregular vascular network in choroid capillary layers and outer retinal layers [36].

### Choroidal metastasis

The choroid is the most common ocular site for metastatic spread, especially for breast and lung cancers. The choroidal metastasis generally appears as creamy white or pale-yellow mass, sometimes associated with overlying SRF. In case of metastases from thyroid carcinoma, renal cell carcinoma and carcinoid tumor the diagnosis may be difficult because they might look orange in color, mimicking a CCH. Nevertheless, metastases are multifocal and bilateral in 15–50% of cases. Unfortunately, the ultrasonographic features are similar in the choroidal metastasis and the CCH. Indeed, the choroidal metastasis occurs as flat or plateau shaped mass with medium to high nonhomogeneous reflectivity and choroidal excavation is present in up to a fifth of cases. Differently from the CCH, the choroidal metastasis is often multilobular with an irregular surface and a diffuse infiltration of the surrounding choroid. [19] FA and ICGA are not conclusive diagnostic exams, but on ICGA the choroidal metastasis displays a larger area of choroidal involvement than on clinical examination or FA [19]. Furthermore, the choroidal filling in FA and ICGA is slower and less intense in both choroidal melanoma and metastasis [17, 42]. The choroidal metastasis is isointense on T1- and hypointense on T2-weighted MRI images, as the choroidal melanoma [39, 40]. On OCT scans, a typical feature is the irregular or “lumpy bumpy” anterior surface of the lesion. Other tomographic elements are SRF with hyperreflective dots, shaggy photoreceptors, alteration of the ellipsoid zone, RPE changes and choriocapillaris compression [43, 44]. On OCTA images, the choroidal metastasis shows lack of flow at the level of the lesion and absence of pathological blood flow in the outer retinal layer, while the CCH displays a richly branched vascular network [19].

### Central serous chorioretinopathy

CSCR is a condition classified among the pachychoroid disease spectrum which typically affects working-age men. A common feature of CSCR is serous retinal detachment, as in the CH. On OCT images, the most distinctive finding of CSCR is SRF, but also pigment epithelium detachments (PEDs), double-layer sign, vacuole sign and hyperreflective dots in the neuroretina and subretinal space are commonly detected. Choroidal vessels dilatation and increased choroidal thickness are visualized with EDI-OCT in patients affected by CSCR. Sobol et al. [45] interestingly observed the same tomographic characteristics in the fellow eye of patients with CCH, suggesting that inherent choroidal changes and lack of proper choroidal vascularization may create a permissive state for the onset of both diseases. Also Kim et al. [46] described greater subfoveal choroidal thickness in the fellow eyes of patients with CCH than in age-matched normal eyes and reported RPE disruptions and/or PEDs in 20% of those patients, suggestive of previous asyntomatic CSCR. For this reason, Kim et al. [46] hypothesized that increased choroidal thickness is associated with risk of developing both CCH and CSCR. On AF, areas of granular hypo-AF and mixed stippled hyper- and hypo-AF are seen in CSCR. FA shows hyperfluorescent areas in all phases and RPE leakage is described with a “smoke-stack” or an “ink-blot” pattern. ICGA visualizes dilated choroidal vessels and choroidal filling defects in the early phase, with mid-phase indistinct hyperfluorescence and late staining [47].

## Treatment

The decision to treat a CCH depends on the presence of symptoms. Indeed, when the CCH is asymptomatic and the diagnosis is incidental, no treatment is required, but close and periodically follow-up is suggested. Conversely, if symptoms occur the treatment depends on the presence of SRF, tumor location and size, and potential visual recovery. Any treatment option aims to the resolution of SRF and the consequent preservation or improvement of VA, while any decrease in tumor size is a gain. In vision threatening CCH, treatment should be started as soon as possible in order to achieve a better visual outcom [1, 48].

Options of treatment include photocoagulation, TTT, radiation therapy, PDT and anti-vascular endothelial growth factor (anti-VEGF) therapy. Available treatment options are summarized on Table 2.

**Table 2:** Available treatment options for CCH

TREATMENT	INDICATIONS	OUTCOME
PHOTOCOAGULATION (Xenon-Argon)	• Limited application • Not indicated in subfoveal CCH and in CCH with extensive overlying SRF because of the left residual scarring	• Poor visual recovery and often temporary SRF resolution • Side effects: left residual scarring. Often need of additional treatments, with a possible damage of the nerve fibers and consequent visual field defects
TRANSPUPILLARY THERMOTHERAPY (TTT)	• CCH with: a. anterior tumor margin posterior to the equator b. Largest tumor base diameter < 10 mm c. Tumor thickness < 4 mm d. Shallow SRF overlying the tumor e. Absence of extensive retinal detachment • Considering TTT after the injection of indocyanine-green (ICG-TTT) with the same indications of TTT	• Early resolution of SRF and slower tumor shrinkage without residual scarring compared to photocoagulation • ICG-TTT: good outcomes in terms of VA improvement, SRF resolution and tumor shrinkage with smaller damage to healthy tissue compared to PDT • CCH previously treated with argon laser photocoagulation or associated with subretinal fibrosis or increased thickness may not show complete regression • Side effects: branch retinal vein occlusion, transient CME, iris atrophy, wedge-shaped visual field defects, pre-retinal fibrosis
RADIATION THERAPY Different types available: a. Episcleral plaque radiotherapy, or brachytherapy; b. Proton beam radiotherapy; c. Lens sparing external beam irradiation (EBRT); d. Gamma knife radiosurgery (GKRS); e. CyberKnife	• Not often considered as first-line therapy • Recommended in cases of extensive retinal detachment or subfoveal location • Useful in those cases where previous treatments failed in reabsorbing SRF	• Good results in terms of SRF resolution, tumor regression and visual improvement in short time also in patients who failed to respond to previous treatments (eg. photocoagulation) • Disadvantages: need of two separate surgical procedures to place and remove the plaque (episcleral plaque radiotherapy). Very expensive and limited availability (proton beam radiotherapy) • Side-effects: radiation-related complications (cataracts, radiation retinopathy, dry eye, and neovascular glaucoma). Headache, nausea, longer treatment time (30–45 min/session) and high cost for CyberKnife
PHOTODINAMIC THERAPY (PDT)	• Ideal first-line therapy for symptomatic CCH with SRF and exudative retinal detachment • Recommended in cases of subfoveal or juxtapapillary location • Ideal candidates: patients aged < 50 years with a pre-PDT BCVA score > 20/200 • Factors predictive of good final visual outcome (≥ 20/40): good baseline VA, smaller tumor size, lack of CME, shorter symptoms duration and lack of treatment before PDT • No standardized protocol yet • Regular follow-up subjects with OCT every 6 months, especially in patients with a marginal detectable tumor on USG following PDT treatment	• Safe, rapid, repeatable, and effective treatment • All published studies reported positive anatomical and functional outcomes with resolution of SRF, shrinkage of the tumor, improvement of VA and need to retreat only in a limited number of cases • Side effects: transient increased SRF accumulation no responsive to anti-VEGF injections, acute exudative maculopathy (PAEM), arteriolar occlusion • Side effects of repeated PDT sessions: choroidal atrophy, transient choroidal effusion and perifoveal hemorrhage
INTRAVITREAL INJECTION OF ANTI-VEGF (Bevacizumab, Ranibizumab) AND DEXAMETHASONE IMPLANT	• Useful in macular edema secondary to CCH • Consecutive Ranibizumab injections in case of CNV after PDT	• Minimally invasive treatment with no effects on the surrounding retinal tissue • Better outcomes in association with other treatments, such as PDT
ORAL PROPRANOLOL	• Rarely used as first or second line-therapy	• Still inconclusive results in literature • Side effects: vision worsening and metamorphopsia
ENUCLEATION	• Last resort treatment for CCH • Often results of the misdiagnosis of a choroidal melanoma. Other reasons: total retinal detachment or neovascular glaucoma	

*TTT* = transpupillary thermotherapy; *EBRT* = Lens sparing external beam irradiation; *GKRS* = gamma knife radiosurgery; *PDT* = photodynamic therapy.

*SRF* = subretinal fluid; *VA* = visual acuity; *OCT* = optical coherence tomography; *CME* = cystoid macular edema; *BCVA* = best correct visual acuity; *PAEM* = acute exudative maculopathy

### Laser Photocoagulation

Xenon arc photocoagulation was the preferred treatment in the 1960s and 1970s, followed by argon laser photocoagulation which came after 1970s. Currently, their application is limited since the visual recovery is poor compared to other therapies and the resolution of SRF is often temporary. Additional treatments are often required, with a possible damage of the retinal structures, especially nerve fibers, producing visual field defects [49–52]. Furthermore, the left residual scarring prevents the use of photocoagulation in subfoveal CCH and in CCH associated with extensive overlying SRF [53].

### Transpupillary thermotherapy

TTT uses a diode laser with a broad beam and a long exposure time. Compared to laser photocoagulation, TTT induces an early resolution of SRF and a slower tumor shrinkage without residual scarring, owing to a heat-induced thrombosis of tumor blood vessels and cytolysis of tumor cells and vascular endothelium. Possible complications of TTT are branch retinal vein occlusion, transient CME, iris atrophy, wedge-shaped visual field defects and pre-retinal fibrosis [54]. TTT can be a primary or a secondary treatment, but CCH previously treated with argon laser photocoagulation or associated with subretinal fibrosis and/or with increased thickness may not show complete regression. The factors just mentioned probably prevent the uptake of heat within the mass, causing the failure of the treatment [55, 56].

Some authors recommend to use TTT to treat CCHs with largest base diameter < 10 mm, tumor thickness < 4 mm, shallow SRF overlying the tumor and absence of extensive retinal detachment [54, 55, 57–59]. When these requirements are not met, other therapeutic options like radiotherapy should be considered [60]. TTT is not indicated in subfoveal CCH for possible side effects on the sensory retina. In these cases, PDT is suggested [61, 62]. Even though juxtapapillary CCH can be managed with TTT, the clinician has to consider that heavy treatment may lead to thermal papillitis or severe nerve fiber defect, but inadequate treatment to avoid these complications may cause treatment’s failure. If a juxtapapillary CCH is treated with TTT, it is recommended to spare the optic disk margin from direct treatment [54].

Some authors [63, 64] evaluated the impact of TTT on CCH after the injection of indocyanine-green (ICG-TTT). As result of both thermal and photodynamic effect, good outcomes in terms of VA improvement, SRF resolution and tumor shrinkage were obtained. Moreover, Tian et al. [63] compared the outcomes of ICG-TTT and PDT and stated that both treatments led to SRF absorption and tumor atrophy, but ICG-TTT shown smaller damage to healthy tissue (hyperplasia or pigment loss and partial vascular occlusion) compared to PDT.

### Radiation therapy

Since the late 1980s, radiation therapy has been a therapeutic option for symptomatic CCHs. This treatment is recommended in cases of extensive retinal detachment or subfoveal location. The three most common types of radiation therapy include: episcleral plaque radiotherapy, proton beam irradiation and lens-sparing external beam irradiation (EBRT).

Commonly, the radiation therapy of choice is episcleral plaque radiotherapy, or brachytherapy, with various radioactive isotopes (Co-60, I-125, Ru-106. Pd-103). It offers good results in terms of SRF resolution, tumor regression and visual improvement in short time (it requires only 2–4 days before the removal of the plaque) [65–68]. Brachytherapy treats the tumor directly from its base and demonstrated to be effective also in patients who failed to respond to previous treatments, for example laser photocoagulation. The main disadvantages are the need of two separate surgical procedures to place and remove the plaque and the risk of radiation-related complications such as cataracts, radiation retinopathy, dry eye, and neovascular glaucoma. For these reasons, radiotherapy is not often considered as first-line therapy.

The newest form of radiation therapy is proton beam radiotherapy. Using charged particles slowed down at the tumor site, it ensures a homogeneous irradiation of the lesion while sparing the surrounding healthy tissue. In literature, there is evidence of retinal reattachment, VA improvement and tumor’s shrinkage with 20 Cobalt Gray equivalent (CGE) [69–72]. Proton beam therapy may be used also in those cases where PDT failed in reabsorbing SRF. However, this procedure is very expensive, has a limited availability and can lead to radiation-associated complications, such as radiation retinopathy, dry eye, cataract and radiation optic neuropathy [69, 70].

EBRT has been adopted to treat DCH with several dose ranges. It affects the entire choroid with a homogenous dose of radiation, giving good outcomes in terms of SRF resolution and VA improvement. One possible side effect is cataract, for this reason a lens sparing technique is recommended. EBRT may be also used in CCH, even if gamma knife radiosurgery (GKRS) guarantees a more precise radiation treatment in a single session.

GKRS with a relatively low marginal dose of 10 Gy was employed both in diffuse and circumscribed CH with positive outcomes [73]. Also CyberKnife represents an effective therapeutic option since it minimizes the irradiation of the nearby critical structures using multiple beams coming from different directions. Disadvantages of CyberKnife therapy include headache, nausea, cataract, longer treatment time (30–45 min/session) and high cost. However, since a single treatment session is usually curative, there is not much difference in terms of treatment cost between PDT and CyberKnife [74].

### Photodynamic therapy

PDT had proved to be a safe, rapid, and effective treatment for CCH, especially in case of subfoveal or juxta-papillary location, where photocoagulation treatment may damage the healthy retina. It uses a photochemical, the verteporfin dye, that selectively occludes CCH’s vascular channels, binding to low density lipoproteins which are largely expressed in the tumor’s endothelium. Following the photochemical injection, a laser is applied, causing a site-specific reaction that spares the choriocapillaris and the sensory retina. For its selectivity, PDT seems to be an ideal first-line therapy for symptomatic CCH with SRF and exudative retinal detachment. Factors predictive of good final visual outcome (≥ 20/40) are good baseline VA, smaller tumor size, lack of CME, shorter symptoms duration and lack of previous treatments [75–77]. Ho et al. [78] stated that the ideal candidates are patients aged < 50 years with a pre-PDT BCVA score > 20/200.

All published studies reported positive anatomical and functional outcomes with resolution of SRF, shrinkage of the tumor, improvement of VA and need to retreat only in a limited number of cases [48, 76, 79–88]. Shields et al. [89] reported that patients treated with PDT obtained significantly better visual outcomes compared to the pre-PDT treatments. Furthermore, CCH treated in the PDT era showed improved tumor regression and better control of CME.

Lo Giudice et al. [90] assessed CCH network after PDT with OCTA and observed that large intralesional vascular channels appeared dark two days after the treatment because of the induced thrombosis. Seven days after PDT, the vascular net reappeared, but slightly less dense than before.

All authors administered an intravenous verteporfin dose of 6 mg/m2 of body surface area, excepted for Lee et al. [91] who compared the standard dose of 6 mg/m2 to a double dose of 12 mg/m2 which provided better tumor regression with similar SRF resorption. Also Byeon et al. [87] compared the therapeutic effect of a modified double-dose PDT with standard-dose PDT and observed a greater reduction in tumor thickness (45.3% vs 20.6%, p = 0.013) and tumor volume (60.0% vs 30.0%, p = 0.006), and a reduced risk of recurrence at 1 year post-treatment.

Some authors reported that infusion was made in a short bolus injection and laser application was started 5 min after the end of the injection [61, 92, 93]. Others used a 10-min infusion and a laser application 15 min after the start of the infusion [83, 94, 95]. Pilotto et al. [96] analyzed the differences between standard and bolus injection in 20 patients randomly assigned using a 1:1 ratio (standard or bolus PDT) and observed a clinical regression of CCH in all cases, with similar VA outcomes, but a reduction in retinal sensitivity (assessed with MP1 Microperimeter, covering 10° centered onto the choroidal lesion) was detected in 70% of bolus PDT patients. Furthermore, 90% of patients who received the bolus showed neuroretinal and RPE changes, precisely reactive RPE hyperplasia (40%), subretinal fibrosis (30%) and macular pucker with retinal traction (20%), whereas only 10% of eyes treated with the standard protocol developed RPE hyperplastic changes.

Pellegrini et al. [97] evaluated efficacy and safety of double fluence PDT (two consecutive spots of 50 mJ/cm^2^ light at 689 nm for 83 s after a dose of 6 mg/m^2^ body surface area over 10 min) in the management of CCH and obtained total reabsorption of macular SRF and no side effects or need for re-treatment.

On the other hand, Kumar et al. [88] assessed safety and efficacy of half-fluence PDT (25 mJ/cm2) in symptomatic peripapillary CCHs and reported a significantly improvement in both VA and central macular thickness, with need of retreatment in 18% patients e no complications, suggesting that also half-fluence PDT may be effective.

The initial treatment protocol also differed. Some authors applied a single spot covering the entire lesion [92, 98], others used a single spot only large enough to cover “the most prominent part” of the tumor [93, 99] and still others applied ≥ 1 overlapping spots or nonoverlapping spots [61, 95, 100, 101]. All authors obtained good results in terms of efficacy and safety, but Stehouwer et al. [93] found an unexpected high recurrence rate of 35% at almost 6 years using a single spot to the thickest area of the hemangioma. Retreatment with PDT of all recurrences was successful. Furthermore, overlapping spots-PDT may be more suitable for large-size lesions and the overlapping part, applied on the most prominent part of the tumor, may ensure the efficacy of the treatment [101].

In the reported cases, the number of treatment sessions ranged from 1 to 5. Although there are no guidelines yet, in most studies retreatment was performed when persistent or recurrent exudation 3 months after the last PDT session occurred [61, 77, 98, 100, 102]. In some cases, treatment interval was abbreviated to 6 weeks [62, 92, 93]. Complete SFR resolution determined treatment interruption. No RPE changes were observed in patients who undergone two PDT treatments, whereas minimal alterations were detected in half of the patients who undergone three PDT sessions [98, 103].

VA gain obtained with PDT resulted to be stable in several long follow-up studies [83, 84] while Stehouwer et al.[93] noted an unexpected high number of recurrences in an 11 years follow-up report. For this reason, they concluded that regular follow-up with OCT every 6 months is recommended, especially in patients with a marginal detectable tumor on USG following PDT treatment.

According to Porrini et al. [98], a complete absorption and shrinkage of tumor vasculature is crucial to prevent the recurrence of subretinal and/or IRF. They suggested that PDT should be repeat until hemangioma is no longer visible on USG. On the other hand, Jurklies et al. [61] reported complete resolution of subretinal and IRF in the macular area as a successful treatment outcome.

Side effects after PDT are rare. Ho et al. [78] described three cases of transient increased SRF accumulation, no responders to injections of anti-VEGF. This phenomenon has been previously reported in patients treated with PDT for intraocular tumors [104, 105] and a possible explanation is the occurrence of vascular thrombosis in the rich vascular bed typical of CCHs. Chakurkar et al. [106] reported a singular case of PDT-induced acute exudative maculopathy (PAEM), successfully treated with an intravitreal injection (IVI) of bevacizumab. Xiao et al. [107] observed a case of retinal arteriolar occlusion occurred two days after a single PDT session in a 33-years old man with no abnormalities at the extensive blood tests. One possible side effect of repeated PDT sessions is choroidal atrophy. In CCH treated with the PDT standard AMD protocol [108], Landau et al. [95] detected a transient choroidal effusion and a perifoveal hemorrhage with subsequent loss of VA, but these side effects were not described by Boixdera et al. [102] who used the same protocol.

### Intravitreal injection of anti-vascular endothelial factor and dexamethasone implant

Some authors [109–114] reported that IVIs of anti-VEGF, such as bevacizumab or ranibizumab, or dexamethasone implant may be useful in macular edema secondary to CCH. A reasonable explanation was provided by Liu et al. which found higher levels of VEGF-A and IP-10 in CCH patients (16 eyes) compared to normal controls (15 eyes) [115]. IVIs of anti-VEGF are minimally invasive and demonstrated no effects on the surrounding healthy retinal tissue, but anti-VEGFs therapy in CCH seems to be less efficient than in retinal neovascularizations. Indeed, while retinal neovascularizations are supported by elevated levels of anti-VEGF factors, hemangiomas probably originate from vascular turbulences and the anti-VEGF suppression does not induce tumor regression as it does in neovascularization. For this reason, in CCH better outcomes were obtained combining anti-VEGFs with other treatments, such as PDT. Choroidal neovascularization (CNV) rarely occurs in association with CCH or after its treatment. Cases of CCH patients developing CNV after PDT have been described and managed with consecutive ranibizumab injections, with favorable outcomes also in very young patients [116].

### Oral propranolol

Propranolol is a nonselective β-adrenergic receptor blocker. β-adrenergic receptors are expressed on capillary endothelial cells and regulate endothelium-dependent vasodilatation and angiogenic factors pathways. Therefore, propranolol may induce endothelium vasoconstriction, decrease expression of basic fibroblast growth factor (bFGF), hypoxia-inducible factor 1α (HIF-1α) and VEGF through the RAF–mitogen-activated protein kinase pathway and inhibit endothelial proliferation [117]. Since VEGF and bFGF are involved in infantile hemangiomas growth, propranolol is the first-line treatment in these lesions [118]. Hypnotizing a similar growth pathway between infantile hemangioma and CCH, Sanz-Marco et al. [117] described for the first time a case of CCH unsuccessfully treated with laser photocoagulation and then managed with oral propranolol (120 mg) daily administered for a month. VA improved to 20/20 and foveal serous detachment disappeared, remaining stable after 8 months. Results in literature are still inconclusive, with some cases of treatment success and others of failure. Following report [119] conducted on treatment-naïve patients managed with oral propranolol stated that patients complained of worsening vision and metamorphopsia at the fourth month, which led to stop the β-blocker therapy even though there was no overall deterioration in VA or aggravation of lesions compared with the beginning of treatment.

## Treatment comparisons

Few studies compared the outcomes achieved with different treatment modalities in the management of symptomatic CCHs.

Scott et al. [52] examined the differences between PDT (n = 5) and laser photocoagulation (n = 23) and described a complete resolution of SRF in all patients managed with PDT versus 57% of patients treated with laser photocoagulation, and better VA outcomes in PDT patients.

Papastefanou et al. [120] confronted PDT (n = 16) versus EBRT (n = 23) and plaque radiotherapy (n = 3) and noted no difference in tumor thickness reduction or VA gain, but PDT shown no side effects, while radiation-related complications were reported.

Mathis et al. [121] compared PDT versus proton beam therapy (PBT) and found that final VA and anatomical outcomes did not differ significantly between the two groups for CCH ≤ 3 mm, although PDT sometimes required multiple sessions. For lesions > 3 mm, PBT seemed preferable because it may treat the tumor in only 1 session with better functional and anatomical outcomes.

Lee et al. [15] evaluated absorption of SRF associated with CCH and found that PDT was more successful in terms of SRF reduction compared to TTT and intravitreal bevacizumab injection. In the same study, PDT was the only valid method for the resolution of CME [15]. In a study by Tian et al. [63] comparing ICG-TTT and PDT, ICG-TTT resulted to be more effective than PDT in terms of VA increase and tumor diameter/thickness decrease. Gunduz et al. [16] reported no significant differences between TTT, ICG-TTT and PDT groups regarding SRF persistence and tumor thickness decrease. On the other hand, final VA at 18 months seemed to be better in the PDT group, but probably only because of the small sample.

In order to understand structural changes following PDT and TTT, Raval et al. [122] compared OCT and OCTA images of 16 eyes treated with either multiple sessions of PDT with/without anti-VEGF injection or TTT, depending on the tumor location and presence of macular SRF. Post-treatment OCTA images showed complete loss of choriocapillaris and absence of deeper choroidal vessels in all TTT-treated eyes, while in the PDT-treated eyes no loss of choriocapillaris and persistence of deeper choroidal vessels were observed. In addition, in patients treated with PDT the tumor size reduced without fibrosis, whereas flat scar with fibrosis and choroid thinning were described in all patients treated with TTT. Considering the tumor reduction without choroidal vessels damage obtained with PDT, Raval et al. [122] suggested that PDT may be the first-line therapy for CCH, particularly in those cases where the lesion involves the macula and the papillo-macular bundle.

## Conclusions

CCHs are benign vascular hamartomas, difficult to differentiate from other choroidal tumors in some cases. Clinical presentation as well as the use of diagnostic tests like USG A/B scan and FA/ICGA are essential to proper diagnose this condition. SD-OCT is mainly used in monitoring CCHs complications, such as CME or SRF, while OCTA represents a promising non-invasive exam for the diagnosis and follow-up, allowing an adequate visualization of both choroidal and tumor vascularization. Nowadays, ICGA is still the most pathognomonic diagnostic test. Despite its benign status, CCH may lead to persistent visual impairment. For this reason, symptomatic patients require an early and proper therapy in order to resolve SRF and improve VA. PDT is the treatment of choice in symptomatic hemangiomas, especially in CCH with subfoveal or juxtapapillary location where other treatments like laser photocoagulation and TTT may cause retinal damages. PDT demonstrated high success in SRF reabsorption with minimal complications and limited cases of retreatment, although there is no standardized protocol yet. In cases of large hemangiomas with extensive retinal detachment, radiation therapy may be preferred. Radiotherapy is not the first-line therapy due to its cost, limited availability and radiation-associated complications. Nevertheless, PBT may be used in those cases where PDT failed and for tumors > 3 mm. Further studies are necessary to identify multimodal imaging findings which may help the clinician to correctly diagnose the CCH and to evaluate the best timing and modality of treatment.

## Search methodology

We conducted a comprehensive literature review using PubMed. The search terms used were “choroidal hemangioma”, “circumscribed choroidal hemangioma”, “choroidal tumors”, “choroidal hemangioma diagnosis”, “choroidal hemangioma imaging”, “choroidal hemangioma treatment”. A thorough search of peer-reviewed articles was conducted. A few articles published before 1990 are included for historical purposes and the lack of research studies on the topic, but the review is based mainly on articles published in the past twenty years. All searches were limited to the English language. Specifically, we were interested in the clinical presentation, multimodal imaging, differential diagnosis and treatment. This review included retrospective studies/series and other reviews. We included case reports only if they contributed new information about characteristics, diagnosis or treatment of the circumscribed choroidal hemangioma.
